# Bone Replacement Materials and Techniques Used for Achieving Vertical Alveolar Bone Augmentation

**DOI:** 10.3390/ma8062953

**Published:** 2015-05-27

**Authors:** Zeeshan Sheikh, Corneliu Sima, Michael Glogauer

**Affiliations:** 1 Matrix Dynamics Group, Faculty of Dentistry, University of Toronto, Room 221, Fitzgerald Building, 150 College Street, Toronto, ON M5S 3E2, Canada; E-Mail: michael.glogauer@utoronto.ca; 2 Department of Applied Oral Sciences, The Forsyth Institute, 245 First Street, Cambridge, MA 02142, USA; E-Mail: csima@forsyth.org

**Keywords:** vertical bone augmentation, alveolar ridge augmentation, biomaterials, bone graft materials, bone replacement, osteoconduction, osteoinduction, creeping substitution

## Abstract

Alveolar bone augmentation in vertical dimension remains the holy grail of periodontal tissue engineering. Successful dental implant placement for restoration of edentulous sites depends on the quality and quantity of alveolar bone available in all spatial dimensions. There are several surgical techniques used alone or in combination with natural or synthetic graft materials to achieve vertical alveolar bone augmentation. While continuously improving surgical techniques combined with the use of auto- or allografts provide the most predictable clinical outcomes, their success often depends on the status of recipient tissues. The morbidity associated with donor sites for auto-grafts makes these techniques less appealing to both patients and clinicians. New developments in material sciences offer a range of synthetic replacements for natural grafts to address the shortcoming of a second surgical site and relatively high resorption rates. This narrative review focuses on existing techniques, natural tissues and synthetic biomaterials commonly used to achieve vertical bone height gain in order to successfully restore edentulous ridges with implant-supported prostheses.

## 1. Introduction

**Figure 1:**
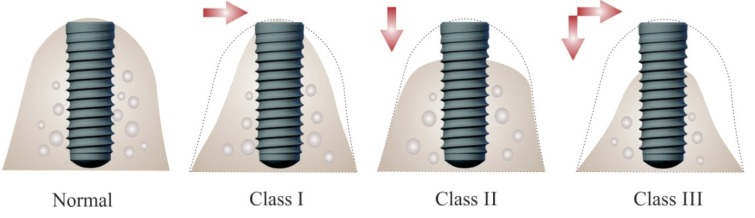
Bone volume insufficiency for implant placement. In Siebert class I ridge defects there is horizontal bone loss with adequate height, which leads to insufficient bone volume for successful placement of regular diameter implants. In class II there is vertical bone loss with adequate width, which leads to insufficient bone volume for proper positioning of regular length implants in correct prosthetic corono-apical position. In class III there is vertical and horizontal bone loss that prevents placement of successful implants in all spatial dimensions.

Advances in biomaterials research and development of new and improved surgical techniques and armamentarium have resulted in an ever increasing use of dental implants for tooth replacement. The long-term success of dental implants is highly dependent upon the degree of osseointegration in sufficient and healthy bone [1,2,3,4,5,6]. Bone volume is often reduced due to extended time after tooth loss before implant placement, or due to periodontitis or trauma [1,7,8]. After tooth extraction an average alveolar bone loss of 1.5–2 mm (vertical) and 40%–50% (horizontal) occurs within 6 months [9,10]. Most of alveolar dimensional changes occur during the first 3 months [11]. If no treatment to restore the dentition is provided, then continued bone loss occurs and up to 40%–60% of ridge volume is lost in first 3 years [12,13,14]. The loss of vertical bone height leads to great challenges to dental implant placement due to surgical difficulties and anatomical limitations [1] (Figure 1). This lack of sufficient bone volume and height if unresolved eventually proves to be detrimental to the final treatment outcome with respect to implant success and survival [1,15].

Various surgical techniques and biomaterials have been developed to make possible the successful placement of dental implants in resorbed alveolar bone [16,17,18,19,20]. Multiple bone grafting techniques, natural and synthetic graft materials have been tested for this purpose [21,22,23,24,25]. Although animal experiments have reported promising results, vertical bone augmentation procedures experience a high rate of failure in clinical practice [21,22,23,25]. The main reasons for failure are poor bone augmentation as a result of soft tissue encleftation and graft shrinkage due to poor blood supply. Granulation tissue formation and lack of adequate bone callus formation are generally caused by graft instability, exposure of graft material to the oral environment and infection [23,24]. Insufficient or delayed vascularization of the graft often leads to a mismatch between blood flow and bone resorption/formation coupling, which can result in unpredictable bone augmentation. In this review article we discuss the various techniques and materials currently available to achieve vertical alveolar bone augmentation.

## 2. Principles of Bone Regeneration

### 2.1. Basic Multicellular Units

The central anatomic structures involved in bone regeneration are the basic multicellular units formed by temporary assembly of osteoblasts (bone forming cells) and osteoclasts (bone resorbing cells). While the two cell types function as a unit in rebuilding the bone structure, they develop from two separate embryonic lineages: osteoblasts derive from mesenchymal stem cells (bone marrow stromal stem cells) while osteoclasts derive from hematopoietic progenitors (monocyte lineage). Two transcriptional factors expressed by osteoblasts, Runt-related transcription factor-2 (Runx2) and Osterix/SP7, are necessary for commitment of mesenchymal progenitor cells to osteoblast lineage [26,27]. The novel zinc finger-containing transcription factor osterix is required for osteoblast differentiation and bone formation [27]. Key factors involved in osteoblast differentiation include vitamin D3, estrogen, parathyroid hormone, fibroblast growth factors (FGFs) and transforming growth factor beta family (TGF-β) [28,29,30]. Osteoclast differentiation depends on activation of colony-stimulating factor-1 receptor/macrophage–colony-stimulating factor/CD115 (MCSF, a colony-stimulating factor receptor) and receptor activator of nuclear factor kappa-B (RANK) receptors [31]. Osteoblasts produce RANK ligand (RANKL) and its high-affinity decoy receptor, osteoprotegerin, to regulate osteoclast differentiation and activation. Therefore, osteoblasts are required for osteoclast differentiation primarily through regulating the balance between RANKL and osteoprotegerin [32].

### 2.2. Space Maintenance

In the oral cavity two specific local aspects impose difficulties in creation and maintenance of space where bone regeneration is intended. One is the pattern of bone loss that often generates non self-containing bone defects covered by soft tissues, muscles and/or prostheses that would collapse onto a grafting site if not supported. The second is the relatively high turnover rate of soft tissues during oral mucosal healing, which would take over the space for bone regeneration if barriers were not used [33]. Therefore, in large defects barrier membranes are used in combination with graft materials to allow for migration of osteoblasts and ingrowth of blood vessels from adjacent osteogenic tissues. To increase mechanical support and stability of membranes, tenting screws, titanium-reinforced membranes or titanium meshes are used in conjunction with graft materials. The relatively slow rate of graft resorption also contributes to space maintenance.

### 2.3. Osteogenesis

A requirement for bone regeneration is the presence or recruitment of osteoblast precursors and growth factors at sites of augmentation. Osteoblast precursors can be provided by the graft material (cancellous autogenous grafts) or by the recipient bed. Growth factors come from the graft, recipient bed and vasculature. It is believed that intramarrow penetration of recipient beds favors both cellular and growth factor migration into the sites where bone is regenerated, which is associated with up to 30% greater bone regeneration and higher bone density at grafted sites [34,35]. Osteoprogenitor cells from the host infiltrate the host within 7 days. Surface osteocytes from cancellous autografts survive and are nourished by diffusion [36]. Similarly, oral and iliac cancellous autogenous grafts were shown to be osteogenic in vertical periodontal defects [37,38].

### 2.4. Osteoconduction

The early phase of bone regeneration at grafted sites is dominated by active bone resorption and formation throughout the graft. The latter phase of incorporation is characterized by osteoconduction and a process known as creeping substitution [39]. Osteoconduction is a function of a bone graft that provides a tridimensional scaffold for ingrowth of host capillaries and osteoprogenitor cells [40]. Many of the bone graft materials used today are able to contribute to new bone formation through this biological process [41]. Material structure and design are critical for osteoconduction. Materials that best mimic bone chemistry are optimal for cellular osteogenic differentiation. Further, macroporosity and patterns of pore interconnection have a significant impact on potential for osteoinduction. High porosity levels are required for blood vessel ingrowth and bone matrix deposition. Pore shape and interconnection size may be limiting factors for vascular flow [42]. Therefore, material composition and design need to allow for finely tuned graft resorption and conduction for new bone formation.

### 2.5. Osteoinduction

**Figure 2:**
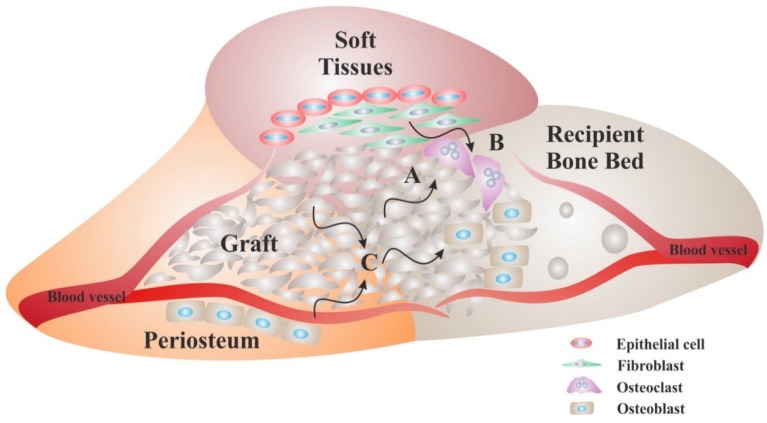
Biological requirements for bone regeneration. Surgical procedures for ridge augmentation are designed based on biological principles of bone regeneration. First, space-maintenance where new bone formation is needed is achieved by use of grafts and/or membranes. In order for bone formation to occur, grafts need to be osteoconductive acting as a scaffold onto which bone resorption and deposition occurs. Most graft materials allow for their resorption by osteoclasts prior to bone deposition by osteoblasts (**A**). Since the turnover rate of soft tissues is higher than that of bone, grafts are used alone when their surfaces have low resorption rates, or in combination with membranes that separate the graft from soft tissues, when their surfaces have high resorption rates; This approach ensures that soft tissues are prevented from occupying the space where bone formation is intended (**B**); Bone deposition by osteoblasts is facilitated by adequate blood flow through the graft and osteoinductive properties of the graft that provide the growth factors necessary for osteoblast differentiation and function (**C**). Some grafts (autologous) act as osteogenic materials when they contain sufficient amount of osteoblasts precursors and growth factors.

The osteoblast precursors differentiate into mature osteoblasts under the influence of osteoinductors and synthesize new bone during the first weeks. Growth factors involved in bone formation act on fibroblast and osteoblast proliferation, extracellular matrix deposition, mesenchymal cell differentiation and vascular proliferation (Figure 2). The complexity of bone induction regulation is reflected by the growth factor specificity of action during early phases of bone regeneration [43]. Platelet derived growth factor (PDGF) and fibroblast growth factor (FGF) act on early stages of induction by stimulating fibroblast and osteoblast proliferation. Similarly, insulin-like growth factor (IGF) acts on cell proliferation but also on extracellular matrix deposition. By contrast, bone morphogenetic proteins (BMPs) act primarily on later stages of osteoinduction such as mesenchymal cell differentiation and vascular proliferation. Transforming growth factor beta (TGF-β) acts on cellular proliferation, matrix deposition and vascularization but not on mesenchymal cell differentiation.

## 3. Techniques for Vertical Bone Augmentation

**Figure 3:**
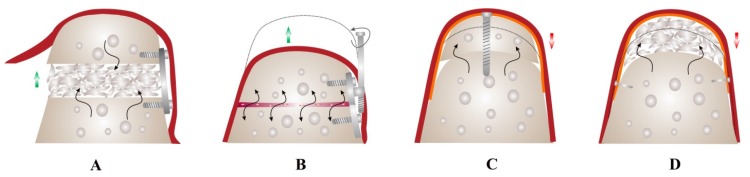
Vertical bone augmentation techniques. Ridge augmentation for implant site development can be achieved through several techniques based on basic principles of bone regeneration, availability of tissue at recipient site and desired clinical outcomes. Osteoperiosteal flaps (OPF) (microanastomosed free bone grafts or interpositional bone grafts) (**A**) and distraction osteogenesis (DO) (**B**) can achieve high bone volume gain (green arrows) but require adequate local tissues at recipient site and are highly technique-sensitive. DO offers the advantage of increasing both soft and hard tissues simultaneously, without the need for grafting; Block grafting (**C**) and guided bone regeneration (**D**) can be used to correct ridge defects of varying degrees but are associated with high resorption rate (red arrows) compared to OPF and DO. This is due to the limited vascularization of the graft and reduced osteogenic surface in the recipient bed (black arrows) compared to OPF. Red, soft tissues; Orange, resorbable or non-resorbable membrane.

Ewers *et al.* proposed a new classification of bone augmentation techniques based on vascularization or induction of vascularization in the graft: class I, microanastomosed free bone flaps; class II, distraction osteogenesis; class III, pedicled segmental osteotomies; class IV, bone morphogenetic induction grafts; class V, nonvascularized bone grafts. The latter was subdivided into class Va-onlay bone grafts and class Vb–guided bone regeneration [44]. There are 4 major recognized techniques for achieving vertical bone augmentation (Figure 3).

### 3.1. Osteoperiosteal Flap Techniques

The osteoperiosteal flap (OPF) is achieved through a vascularized segmental osteotomy performed on alveolar bone. The biologic principles of osteoperiosteal flap techniques are based on vascularization studies and experience with Le Fort I techniques in craniomaxillofacial surgery. Alveolar bone receives blood supply form both bone marrow and periosteum, the latter becoming more significant with age when atrophy of the ridge is associated with decreased bone marrow blood flow. This technique depends on maintenance of vascularization in bone fragments from periosteum. Osteoperiosteal flaps through segmental osteotomies are used in combination with interpositional grafts in the gap generated by transposition of the flap in the desired position to achieve vertical ridge gain.

The micro-anastomosed free bone flaps such as fibular grafts are used in craniomaxillofacial surgery to correct severe bone deficiencies. These grafts offer bone gain of the highest quality (native bone) combined with greatest bone volumetric stability due to continuous blood flow achieved through venular and arterial microsurgical anastomosis [45,46]. However, free bone grafts are extremely technique-sensitive and are associated with significant morbidity at the donor site compared to other techniques used for implant site development. Therefore free bone flaps are reserved for reconstruction of severe mandibular deficiencies due to trauma, cancer of dysplasia. Numerous studies reported successful and stable results with fibular free grafts used for implant site development [47,48].

OPF combined with interpositional (inlay) grafts are increasingly being used more for implant site development in ridges with height deficiencies. The main advantage of osteotomy-based techniques is the preservation of the attached gingiva and even the papillae in some cases [49,50]. Sandwich techniques are similar to distraction osteogenesis in terms of surgical approach and having similar healing patterns and end results [51,52]. As a replacement to callus distraction, a gap is formed by the placement of the bone fragment in final position and stabilized and fixed with either osteosynthesis screws or the dental implant itself [49,53].

### 3.2. Distraction Osteogenesis

Distraction osteogenesis (DO) is a technique used in craniomaxillofacial surgery to achieve high bone volume gain in all spatial dimensions. DO is based on the biological principle of bone callus mechanical elongation through slow and progressive separation under tension of two bone fragments surrounding the callus to achieve new bone formation [54,55,56,57,58,59,60,61,62,63,64,65]. The DO technique includes three phases: (i) the latency phase of 7 days, when soft tissues heal around the surgical site where the distractor is placed; (ii) the distraction phase, when the two bone fragments are separated incrementally at a rate of 0.5–1 mm/day; and (iii) the consolidation phase, when the newly formed bone mineralizes and matures [65,66,67,68,69].

The potential of DO to achieve significant vertical bone augmentation has been extensively reported in the literature [70,71,72]. Devices utilized for DO can be of intraosseous or extraosseous configuration [73,74,75,76]. Intraosseous distractors have been tested in dogs and demonstrated gains in vertical height of bone up to 9 mm [56,72]. An intraosseous approach with a small-diameter device has been tried and achieved vertical bone augmentation of 9 mm [74,77]. One intraosseous type of DO device used for alveolar bone distraction is the distraction implant. This device allows for simultaneous placement of the future dental implant (the apical distraction cylinder), thus reducing the numbers of surgical procedures needed for tooth replacement. A partially biodegradable distraction implant is currently being tested in dogs [78]. However, adequate bone width and proper distraction vector control are required for success with use of such devices.

Extraosseous distraction systems anchored to the cortical plate are more commonly used than intraosseous devices [62,79,80]. 4 to 6 mm of vertical height gain has been reported with prosthetic restorable distracters [81]. DO can achieve significantly greater and stable bone height gain compared to other vertical augmentation techniques and implant therapy in distracted bone shows favorable long term results [54]. However, there is evidence of higher rate of complication associated with DO [82,83,84,85,86]. One of the major problems encountered with DO is vector control, particularly against muscle pull. This often leads to lingual inclination of the transport segment in the mandible. Vector control is one of the most critical aspects of DO. Other potential complications include inability to mobilize the transport fragment, interference of the distractor with occlusion, distraction prevented by premature consolidation, loss of the distractor, infection, perforation of the mucosa by the transport segment, dehiscence of incision, fracture of the mandible and resorption of the transport fragment [87]. DO may allow for greater vertical regeneration from native bone, but it is of little use in routine clinical practice due to sensitivity of the technique and strict anatomical requirements for predictable results [19].

### 3.3. Block Graft Techniques

To increase the vertical height of mandibular and maxillary edentulous ridges, onlay grating using bone blocks was first introduced in early 1990s [88]. The classic block augmentation technique involves the use of an autologous bone block fixed to the recipient ridge with osteosynthesis screws or dental implants [21,89,90,91,92,93]. After performing recipient site corticotomy to encourage blood clot formation and bone marrow osteoblast precursor migration into the graft, the latter is laid over the defective recipient bed devoid of soft tissues, and immobilized. The most common donor sites for block grafts are the mandibular ramus or mental region (intraoral) and the iliac crest (extraoral) [94,95,96,97,98]. Several autologous bone grafting techniques have been used for the treatment of severely resorbed edentulous mandible and maxilla [99,100,101]. In particular, the use of barrier membranes for block grafts seems to significantly improve the clinical outcome [102,103,104,105,106,107].

Extraoral autogenous bone harvested from the iliac crest was used to gain ridge height with varying degrees of success, the main disadvantage being the high resorption rate before implant placement and after loading [94]. This may be due to low cortical to trabecular ratio in the graft, memory of endochondral *vs.* intramembranous ossification and different osteoblast mechano-sensing memory between donor and recipient sites. Less common extraoral donor sites for block grafts used in ridge augmentation include the tibia and cranial vault [108,109]. The use of such autologous grafts is not common in practice mainly due the aforementioned disadvantages and the high morbidity associated with the donor site.

Intraoral block grafts are commonly harvested from the mandibular ramus or the symphysis, the latter offering the greatest bone volume [23,110]. However, ramus grafts are associated with significantly lower morbidity compared to symphysis grafts that may be associated with significant post-operative pain, altered sensation of mandibular anterior teeth, neurosensory disturbances in the chin region, temporary mental nerve paresthesia and mandibular fracture [111,112]. Therefore, the symphysis is often reserved for cases that require thicker block grafts that cannot be obtained from other intraoral donor sites.

Although onlay bone grafting procedures have reasonably acceptable results with an improvement from the initially reported 50% failure [101], complications are often observed at the donor site [113,114,115,116,117,118]. Further, implant survival rates continue to remain a concern for full-arch onlay grafting procedures [113,114]. The close contact and stabilization of autologous block grafts to the recipient bed is considered crucial towards achieving successful clinical results [119,120]. This can be assured by simultaneous implant placement [88,101,121,122], or the use of fixation screws [104,123]. A study on 115 autologous block grafts reported only one failure when all these conditions were met [109]. Revascularization and rate of remodeling can also be enhanced through decortication of the recipient bed, and inlay shaping [34,109,120,121,124,125]. Ridge augmentation with allograft block material has also demonstrated success [126,127,128]. However, histologically the performance of these allogenic grafts remains inconclusive.

### 3.4. Guided Bone Regeneration

**Figure 4:**
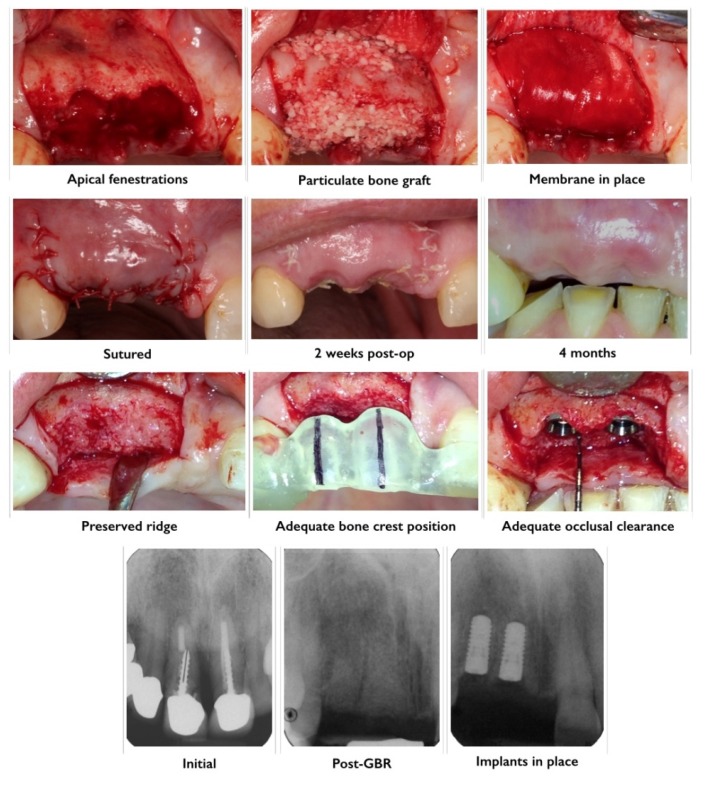
Alveolar bone preservation using a particulate allograft material. Exodontia of teeth 1.1 and 1.2 (failed endodontic treatments, post fracture, loss of clinical attachment) was performed and full thickness buccal and palatal flaps were raised. Post-extraction sockets were generously irrigated with saline and inspected for integrity of bony walls. Apical fenestrations of the buccal wall were detected at 1.1 and 1.2 sites. Irrigated particulate freeze dried bone allograft (FDBA) was gently packed into sockets and covered with a double-layered porcine collagen membrane. Post-guided bone regeneration (GBR) healing was uneventful. Four months post-GBR adequate height and width of bone was observed clinically and radiographically at 1.1 and 1.2 sites. There was adequate distance between alveolar crest and cement-enamel junction of adjacent teeth as well as occlusal clearance for the future crowns.

Guided bone regeneration (GBR) is a technique that works on the principle of separating particulate graft material from surrounding soft tissue to allow for bone regeneration, which occurs at a slower rate compared to soft tissues [129,130]. Resorbable (usually collagen based) or non-resorbable (usually expanded-polytetrafluoroethylene based) membranes are frequently used to stabilize the graft material, limit graft resorption and act as an occlusive barrier toward the surrounding soft tissue regeneration and infiltration [130] (Figure 4). The desired clinical outcome and knowledge of the local anatomy, graft type used and biology of healing drive the choice of a specific membrane. The main problems associated with particulate graft techniques are the higher than anticipated graft resorption rate and the anatomical limitations for graft containment [131,132].

The principles of GBR were initially applied for implant site development in atrophic jaws [133]. The expansion of GBR to a large variety of bone defect types led to the widespread use of this technique in clinical practice [9,134,135]. In some cases, the use of barrier membranes is not warranted and the graft material can be used alone to fill the defect [136]. This is commonly referred to as bone replacement graft. Acceptable predictability of bone gain with use of such grafts is limited to small self-containing defects. Bone resorption has been reported with the use of autografts without membranes [123,137]. Therefore, membranes are utilized in non-space making bone defects that require space maintenance and prevention of soft tissue ingrowth where bone regeneration is required [18,136,138]. In most cases, the use of membranes alone for GBR is associated with membrane compression into the defect space by overlying soft tissues [139,140,141,142]. In a study it was shown that significantly less resorption of block grafts was observed when they were used in combination with expanded-polytetrafluoroethylene (ePTFE) membranes [102]. Barrier membranes combined with particulate and/or block grafts materials have resulted in more predictable clinical outcomes [133,138,141].

Treatment of complex vertical defects requires a stable and stiff membrane, usually made of titanium or metal-reinforced polytetrafluoroethylene (PTFE) [18,131]. GBR therapy by means of titanium reinforced non-resorbable barrier membranes in conjunction with dental implants has been carried out with varying levels of clinical success [1,58,133,143,144,145,146,147]. Vertical GBR is a sensitive technique that limits clinical success, and failure often occurs due to wound dehiscence [1,58,144,147,148,149]. Another limitation of vertical GBR is the ability to regenerate bone along the long axis of the applied force [1,54,55,56,57,58,60,61,62,80,92,93,150,151]. Specific problems associated with titanium membranes used for GBR are fibrous ingrowth through wide holes present in their structure and exposure of the membrane [107,152].

### 3.5. Minimally Invasive Approaches to GBR

The concept of minimally invasive surgery was devised to achieve vertical bone regeneration and to prevent post-operative complications and graft exposure [153,154,155,156,157,158,159,160,161,162,163,164]. A subperiosteal tunneling technique was developed in late 1970s by Kent *et al.* [154]. This technique involved a small surgical incision made in the alveolar ridge to elevate the periosteum and inject a low viscosity paste of hydroxyapatite (HA) particles [154]. Although appealing, it was found that HA particles were unstable and diffused into adjacent tissues causing the formation of a fibrous capsule that prevented bone formation [160,161]. Newer graft materials with optimized viscosity and an improved surgical technique continued to offer potential for this method but results are controversial [153,154,155,157,158,159,160,161,165].

There is still insufficient comparable quantitative data to assess the clinical usefulness of this technique. However, some studies demonstrated that tunneling combined with screw or membrane mediated stabilization of the grafts can be a predictable vertical augmentation technique [153,154,155,156,158,159,160,161,162,163,165,166,167]. Calcium phosphate based biomaterials such as brushite cement pastes have been evaluated in various *in vivo* studies as injectable pastes with controlled viscosity and additives to achieve minimally invasive vertical bone augmentation [168,169,170,171,172,173,174,175,176,177].

## 4. Natural Transplants and Synthetic Bone Replacement Graft Tissues and Biomaterials

Materials used for bone augmentation are divided into natural transplants (autografts, allografts and xenografts) and synthetic materials (alloplasts) [7,8,178] (Table 1). These graft materials are used for clinical applications based on the hypothesis that they are osteogenic, osteoinductive, osteoconductive or possess a combination of these properties [17].

**Table 1:** Bone replacement graft tissues and materials.

Human bone sources		Non-human natural sources	Synthetic sources (Alloplasts)	
Autografts	Allografts	Xenografts	Bioactive glasses	Bioceramics
-Extra oral sites	-Fresh frozen bone	-Bovine Hydroxyapatite	–	-Hydroxyapatite
-Intra oral sites	-Freeze dried bone allograft (FDBA)	-Coralline calcium carbonate	–	-Other calcium phosphates (Tricalcium phosphate, brushite, monetite)
–	-Demineralized freeze dried bone allograft (FDBA)	–	–	

### 4.1. Autografts

Autografts are harvested and transferred from intraoral or extraoral site to bone deficient-sites within the same individual. Autografts are the most predictable osteogenic organic graft for osseous tissue regenerate [179,180,181]. Grafts harvested from the iliac crest provide optimal osteoconductive, osteoinductive and osteogeneic properties [182]. However, due to the donor site morbidity, increased cost and graft volume limitations, the use of other graft materials became more common in clinical practice [182,183,184,185,186] (Table 2). It has also been noted that autografts may fail at recipient sites as the majority of cellular (osteogenic) elements do not survive the transplantation procedure [187].

**Table 2:** Advantages and disadvantages of using autografts for bone augmentation.

Advantages	Disadvantages
Biocompatible	Need for additional surgery to procure the tissue
Osteoinductive	Increase in operative time and cost
Osteoconductive	Donor site morbidity and postoperative pain
High osteogeniec potential	Increased risk of fracture to donor site
Adequate mechanical strength	Limited amount of tissue can be procured
Available in both cortical and cancellous types	High variability in quality of harvested bone tissue

The most common sites for autograft harvesting are the mandible (chin, mandibular ramus, and mandibular corpus) [98,188], the maxilla (tuber, spina nasalis, and crista zygomatico-alveolaris), the calvaria, the iliac crest (pelvic rim) and the tibia [98]. There is less morbidity associated with intraoral donor sites when compared to extraoral sites [95]. The mandibular bone is the most common transplant in dental surgery [95] and the graft may be harvested as bone blocks or milled to generate particles [95,132]. A scraper can also be utilized to obtain bone chips [132]. The most common extraoral donor site to harvest large amounts of autologous cortical-cancellous bone is the pelvic rim [189].

Autografts can be of cancellous or cortical nature or a combination of both. Cancellous grafts have the ability to revascularize sooner than cortical grafts due to their spongy architecture. Revascularization of these grafts begins around the fifth day after transplantation [190]. The cortical grafts have a high initial strength that decreases over time. After several weeks to 6 months post implantation, cortical autografts have been shown to be 40%–50% weaker than normal bone when strength is compared [190]. Conversely, cancellous autografts are softer initially because of their porous (open) architecture. Over a period of time they continue to gain strength and biomechanical stimulation is critical for achieving adequate dimensional stability and strength [17]. Vertical and horizontal bone augmentation with block or particulate autografts in combination with GBR shows that both techniques are clinically successful for implant placement [107,131,191,192]. The histological outcomes, including revascularization and bone remodeling, of the two techniques differ with block grafts outperforming particulate grafts in terms of bone-to-implant contact and bone fill values [191].

### 4.2. Allografts

Allografts are harvested from genetically non-identical members of same species. There has been a great interest in allograft use for implant site development since availablility in large quantities and use off-the-shelf eliminate the drawbacks associated with a second surgical site. Although allografts undergo multiple treatments prior to use, risk of disease transmission is still a possibility. It has been estimated that the risk of human immunodeficiency virus (HIV) transmission is 1 in 1.6 million with use of allografts [193]. There have been some reports of cross-infection and incidences of disease transmission of bovine spongiform encephalopathy [194,195,196]. However, these very low risks are far outweighed by the advantages associated with allografts, which resulted in an increased use of allografts in routine practice [166,197,198,199,200]. Allograft particles of different sizes and origins (cortical, cancellous or both) are being used for various bone augmentation procedures such as ridge augmentation [147,166,200,201], sinus augmentation [122,124,202,203,204] and in ridge preservation after tooth extraction [201,205,206,207,208,209,210]. Allografts are mostly prepared as fresh, frozen, freeze-dried, mineralized and demineralized, and each of these are available as cortical chips, cortical granules, cortical wedges or cancellous powdered grafts [17,211].

#### 4.2.1. Fresh or Frozen Iliac Cancellous Bone and Marrow Allografts

Fresh or frozen iliac cancellous bone and marrow tissues possess the highest osteoconductive and osteoinductive potential among all allografts available [212,213]. Clinical studies in patients having atrophic maxillary ridge were subjected to bone grafting with human block grafts of tibia fresh-frozen chips and histological analysis revealed a living bone that showed features characteristic of mature and compact osseous tissue surrounded by marrow spaces [214,215]. The risk of disease transmission, antigenicity and extensive cross-matching and treatment required has rendered the use of frozen iliac allografts obsolete in modern practice [17].

#### 4.2.2. Mineralized Freeze-Dried Bone Allografts

Freeze-dried bone allografts (FDBA) have been extensively used for treatment of periodontal defects [216,217,218,219,220,221,222,223,224,225]. Compelling evidence suggests that the health risks associated with fresh frozen bone is minimal [226], and clinical success of grafting procedures is high [227]. It is assumed that the process of freeze-drying affects the immune recognition in the host by distorting the three dimensional presentation of the human leukocyte antigens on the surface of allograft particles [226,228]. FDBA possess inferior osteoinductive and mechanical properties when compared to fresh or frozen allografts [17,227]. Cortical FDBA did not elicit an immune response in non-human primates [219]. Advantages of cortical *vs.* cancellous FDBA include higher volume of bone matrix that increases graft resorption time, higher inductive potential through growth factors stored in the matrix and less overall antigenicity. FDBA are considered osteoconductive [40,229], space maintaining and can be used in combination with autografts to enhance the graft’s osteogenic potential [230]. Ridge augmentation procedures using FDBA blocks have demonstrated formation of vital and mineralized bone with lamellar organization at the grafted sites [231,232]. Ridge augmentation results suggest that FDBA in conjunction with resorbable membranes may be an acceptable alternative to the autogenous block graft in the treatment of compromised alveolar ridge deficiencies [126].

#### 4.2.3. Demineralized Freeze-Dried Bone Allografts

Demineralized freeze-dried bone allografts (DFDBA) are frequently used for maxillofacial and periodontal grafting procedures alone or in combination with FDBA or autografts. Compared to other bone regeneration materials DFDBA has the advantage of rapid resorption and exposure of osteoinductive proteins following demineralization [198,233,234,235]. The osteoinductive potential of DFDBA is mainly attributed to morphogenetic proteins (BMPs) stored in the matrix [236,237]. Growth and differentiation factors have been shown to be present in DFDBA preparations [238,239,240,241]. The bioactivity of DFDBA seems to depend on donor age, with grafts harvested from the younger individuals having higher osteogenic potential in comparison with grafts from older individuals [242,243,244]. In maxillary sinus augmentation procedures, DFDBA showed 29% new bone formation while autogenous grafts showed 40% in comparison [245]. It has also been observed that DFDBA particles situated near pre-existing bone were enclosed by new bone, whereas particles located near the center of the graft show no signs of remineralization or new bone formation [245,246]. However, there are reports that show poor bone formation with commercially available DFDBA [240,247].

### 4.3. Xenografts

Xenografts are derived from species other than human. They are considered to be biocompatible with human recipients and have osteoconductive properties. Bone grafting using xenografts in aseptic bone cavities was first reported in 1889 [248]. Xenograft materials have shown potential for resorption and replacement by new bone at recipient sites over time [249,250,251]. Commercially available bovine bone is processed to yield natural bone mineral without the organic component. Anorganic bone of bovine origin comprises of a HA skeleton that retains the microporous and macroporous structure of cancellous and cortical bone [252] remaining after low-heat and chemical extraction of the organic component. In the past, bovine xenografts had failed due to graft rejection [253], which was probably due to chemical detergent extraction techniques that left residual proteins and hence produced adverse reactions [254]. Xenografts of bovine origin carry a theoretical risk of disease transmission. Although existing data indicates a negligible risk of bovine spongiform encephalopathy, concerns still exist [255,256]. An advantage of these graft materials is the higher osteoconductive potential compared with synthetically derived materials. Bovine-derived bone grafts (particulate and blocks) have successfully been used for the treatment of human intrabony defects and ridge augmentation [122,124,250,255,257,258,259]. The bone blocks of xenogenic origin used in vertical bone augmentation studies are very brittle and lack toughness. They often break during the screw fixation process which results in a more sensitive surgical procedure and less than favorable healing process [115,117,248].

Coralline calcium carbonate is obtained from natural coral, genus *Porites*, and is composed primarily of aragonite (>98% calcium carbonate). It has a pore size of 100 to 200 μm which is similar to that present in cancellous bone [260]. The relative high porosity of ~45% provides a large surface area for graft resorption and replacement by new bone [261]. Calcium carbonate is resorbable *in vivo*, unlike HA that can also be derived from the same coral by heat conversion. Calcium carbonate does not require surface transformation to carbonate like other graft materials in order to induce bone formation; hence it can potentially initiate new bone deposition rapidly [260]. Coralline calcium carbonate was also shown to have high osteoconductivity and it does not undergo fibrous encapsulation [262]. Coralline calcium carbonate was associated with a significant gain in periodontal ligament (PDL) clinical attachment, reduction of probing depths and greater defect fill in periodontal regeneration applications [263,264,265].

### 4.4. Alloplasts

Alloplastic bone graft materials are synthetic materials developed to overcome the inherent problems associated with autograft use [266]. The major advantages of alloplastic materials include their high abundance relative to natural materials, no risk of disease transmission and the very low antigenicity [216]. Alloplasts can be manufactured in various forms and with varying physicochemical properties. They can be made available in both resorbable and nonresorbable forms and can be customized with varying levels of porosity and pore sizes [17,267,268]. Alloplastic materials are mainly osteoconductive without intrinsic potential for osteogenesis or osteoinduction and have been used successfully in periodontal reconstructive surgery [216]. The most common alloplastic materials are tricalcium phosphates (TCP) [269], bioactive glasses [270], HA and dicalcium phosphates [271]. One feature that appears to be critical for success with use of alloplasts is the relative rough structure and large particle size, which proved to allow for adequate bone ingrowth [272].

#### 4.4.1. Tricalcium Phosphate

TCP is a porous form of calcium phosphate. TCP has two crystallographic forms, α-TCP and β-TCP [273]. The most common type is β-TCP and it is used as partially resorbable filler that allows for bone formation and replacement [216,274]. Over the years β-TCP has gained high acceptance as a bone filler material despite results not always being predictable. When a direct comparison is made, the allogenic grafts appear to outperform β-TCP in terms of resorption and bone formation [275]. There is evidence that TCP granules undergo fibrous tissue encapsulation and do not stimulate bone growth adequately [104,105]. On the other hand, some studies reported bone deposition with β-TCP [275,276,277]. Vertical and horizontal ridge augmentation using α and/or β TCP has been evaluated in animal and clinical studies with variable results [278,279,280].

#### 4.4.2. Synthetic Hydroxyapatite

Synthetic HA have been marketed for use in variety of forms: porous non-resorbable, solid nonresorbable, and resorbable (non-ceramic, porous) [17,19]. HA is non-osteogenic, not conclusively osteoinductive, but rather functions as an osteophillic and osteoconductive graft material. The resorptive potential of HA is dependent upon the temperature at which it is processed. When prepared at higher temperatures the HA produced is dense, non-resorbable and has a larger crystal size [281]. The dense HA grafts are osteoconductive, osteophillic and act primarily as inert biocompatible fillers. It has been shown that HA can produce a clinical defect fill greater than flap debridement alone in the treatment of intrabony periodontal defects [282,283]. A resorbable HA, which is particulate and porous, processed at low temperatures is also available for clinical use. This type of HA is a non-sintered (not ceramic) precipitate with particles measuring 300 to 400 μm in size. It is believed that this non-sintered HA acts as a mineral reservoir inducing bone formation via osteoconductive mechanisms [284]. The resorption rate is slow allowing grafts to act as a scaffold for bone replacement [285]. Few studies also reported osteoinductive potential with porous HA grafts [286,287], and early implant loading studies in ridges vertically augmented with nano-structured hydroxyapatite has shown promise [143]. Sinus elevation and alveolar ridge augmentation with HA granules alone [288] or in combination with autogenous bone grafts had high success rates [289,290,291,292,293]. Vertical ridge augmentation of atrophic mandible (posterior region) with customized, computer-aided design/computer-aided manufacturing (CAD/CAM) porous hydroxyapatite scaffolds has been tried [294]. However, HA has limited *in vivo* resorption and remodeling capacity, and are therefore unsuitable as onlay bone graft substitutes for vertical bone augmentation [19,295].

#### 4.4.3. Bioactive Glasses

Bioactive glass is composed of silicon dioxide (45%), calcium oxide (24.5%), sodium oxide (24.5%), and phosphorus pentoxide (6%) [270]. When implanted *in vivo* the pH of the local environment increases >10, and a silicon-rich gel is formed on the bioactive ceramic surface with the outer layer serving as a bonding surface for osteogenic cells and collagen fibers [296]. Particle sizes range from 90–710 μm to 300–355 μm [270,297]. Formation of hollow calcium phosphate growth chambers occurs as a result of phagocytosing cells penetrating the outer silica gel layer and resorbing the gel. This leads to formation of pouches where osteoprogenitor cells can adhere, differentiate and proliferate [298]. Several studies reported bioactive glasses to have superior manageability, hemostatic and osteoconductive properties and may act to retard epithelial down-growth [206,296,298,299,300,301,302,303]. Clinical reports of alveolar ridge augmentation with bioglass showed bone formation in intimate contact to the particles [304]. However, since bioglass undergoes no resorption, bone forms around the particles and grows via osteoconduction [305]. This limits the ability of bioglass to work as a bioresorptive scaffold for vertical alveolar bone augmentation.

#### 4.4.4. Dicalcium Phosphates

Recent studies have shown that dicalcium phosphate compounds with high solubility at physiological pH can be successfully utilized for vertical bone augmentation [97,306,307]. Brushite (dicalcium phosphate dihydrate, DCPD), has the ability to support partial osteogenesis that results in generation of varying amounts of woven bone and fibrovascular tissue [169,308]. Brushite cements have been tested for both vertical bone augmentation and bone defect repair as injectable cements or as preset cement granules [306,309]. Injectable brushite cement has been evaluated in animal studies for minimally invasive craniofacial vertical bone augmentation [306]. Brushite cement was injected under the periosteum on the bone surface, allowing it to set and provide enough healing time to promote vertical bone growth in the local area of cement injection [306]. Several clinical studies have also shown that injectable brushite cements are capable of regenerating bone in buccal dehiscence defects, atrophic ridges and maxillary sinus floor elevation procedures [310]. Similarly, preset granules of brushite cement also promoted vertical bone augmentation in animal models [311]. The amount of vertical bone growth obtained with brushite cement granules was higher than that obtained with commercial bovine HA materials *in vivo* [312]. However, brushite cements exhibit limited *in vivo* resorption due to phase conversion to insoluble HA upon implantation [273,313]. This results in incomplete vertical bone growth in the treated site. Development of brushite cements that do not convert to HA after implantation is necessary to allow for clinical application [306].

Monetite (dicalcium phosphate anhydrous, DCPA) based biomaterials resorb at faster rates compared to brushite [314,315,316] and do not convert to HA [312,317]. Monetite bioceramic materials have been tested as onlay blocks (3D printed) for vertical bone augmentation and in the form of granules as bone defect fillers [2,273,307,317]. It has been shown that monetite blocks can be used as synthetic onlay bone grafts and can achieve adequate vertical augmentation for dental implant placement [97]. Monetite granules were able to promote bone healing in post-extraction dental alveolar sockets in human patients [307] and of craniofacial defect in animals [317]. The clinical performance of monetite granules has been compared with commercially available bovine HA, and demonstrated greater resorption *in vivo* and bone formation in the alveolar ridge sockets [307].

## 5. Developments and the Future of Bone Augmentation

The development of biomaterials and techniques for alveolar bone augmentation applications is a challenge from an engineering, surgical and biological perspective. In the field of biomaterials research, degradable materials for bone augmentation are actively sought. Biodegradable natural and synthetic polymers and bioceramics are already in clinical use as for bone tissue engineering applications [318,319,320,321,322]. The degradation rate is one of the most important characteristics for materials to be used for achieving gain in vertical height of bone. Bioceramics show the ability to degrade predictably and show *in vivo* resorption by cell-mediated and solution-driven processes and demonstrate progressive replacement by lamellar true bone [323,324]. The newer generation of biomaterials will have to be fine-tuned in terms of their physico-chemical properties to have more predictable and improved graft resorption after implantation to be used as effective scaffolds for bone augmentation.

Research in the field of bone augmentation and regeneration is currently focused on cellular, molecular and gene therapeutics [325,326,327,328,329]. Bone morphogenetic proteins (BMPs) have generated a lot of interest recently, as they are differentiation factors [330] and have the ability to differentiate osteoprogenitor cells into mineral forming osteoblasts and stimulate vascular proliferation [331]. BMPs have shown promising results for intraoral applications such as sinus augmentation and ridge preservation [259,332,333,334,335]. The most studied BMPs for bone regeneration applications are BMP-2 and BMP-7 [336,337]. Many preclinical studies have shown the usefulness of recombinant human BMPs for the regeneration of bone tissue [338,339,340,341]. Bone defect healing using genetic approach where an implant comprising of a bioresorbable polymer mixed with mesenchymal stem cells transfected with adenovirus BMP-2 has been demonstrated [342]. Systemic administration of rhBMP-2 has been shown to result in increased activity of mesenchymal stem cells [343]. It has been seen that rhBMP-2 when delivered when using a calcium phosphate carrier, a liposome carrier and collagen sponges result in accelerated bone healing in rat and rabbit models [342,344]. Other femoral defect studies in sheep have shown evidence of new bone formation four weeks post rhBMP-2 grafting and histological evaluation after fifty two weeks revealed woven and lamellar bone being formed [345]. Studies in dogs have evaluated use of BMP in bone defects revealed complete healing of mandibular defects within three months and significant improvement was observed in degree of mineralization, bone thickness and biomechanical strength [346]. A great deal of resources has been invested in the area of micro and nanoparticles in search of simple, efficient and affordable drug delivery systems [347,348,349,350]. Researchers have also tested microspheres and nanoparticles for delivery of BMPs, cytokines and mesenchymal cells [351,352,353,354]. Following promising results of poly-lactic-glycolic acid (PLGA) based delivery systems; microspheres of PLGA have been studied in various animal models including calvarial bone defects in rats [355], rat femur [356] and rabbit calvarial defects [357].

Platelet derived growth factor (PDGF) has also shown great potential for use in bone regeneration [358]. Recombinant human PDGFF-BB (rhPDGF-BB) and inorganic bone blocks have been tested for vertical bone augmentation and demonstrated increased vertical gain compared to controls [359]. PDGF used in combination with ePTFE membranes around implants in dogs was shown to induce rapid and increased bone formation around implants compared to no-PDGF control [358]. Vertical bone augmentation using collagen membranes and chitosan sponges with PDGF showed promising results [360,361]. However, the optimal dosage and carriers for PDGF are still to be determined and further animal and human studies are necessary before clinical application. A new approach to bone augmentation is separation of platelet-rich plasma (PRP) from patient blood to be added to the graft material [362,363,364,365]. Initial results showed more and denser bone compared to autografts used alone for bone augmentation procedures [362]. Research has shown that PRP *in vitro*, stimulated PDL and fibroblast cell line growths and keratinocyte growth rate was inhibited [366]. A split mouth clinical trial with matched defects of 13 patients found significantly improved results with PRP [367]. The combination of PRP with other graft materials has been inconclusive [368,369]. Gene therapy is based on the principle of modified genetic material being delivered to cells in order to enhance their regenerative potential by increased production and local concentration of differentiation and growth factors [370,371]. A cellular tissue engineering approach is being explored in which the regenerative potential of bone tissue is used by performing *in vitro* amplification of osteoblasts or osteoprogenitor cells grown with and on 3D constructs [372,373,374]. The seeding of constructs with mesenchymal stem cells also holds great promise and can potentially be investigated in future for vertical alveolar bone augmentation therapy [375,376].

Various approaches discussed here separately or in combination have the potential for providing improved tissue regenerative results [370]. It is expected that the next generation of biomaterials will demonstrate vast improvements in graft and biological tissue interfacing based on the knowledge gained from recent research and allow clinicians to achieve more predictable clinical results with regards to vertical alveolar bone augmentation.

## 6. Conclusions

There are a plethora of techniques with various combinations of natural and synthetic graft materials that can be utilized for achieving vertical alveolar bone augmentation. There is no single ideal technique or graft material to choose in clinical practice but rather an increasing number of materials and methods to be used in individualized approaches to ridge reconstruction. Treatment protocols that involve less invasive, more reproducible and less technique sensitive vertical bone augmentation procedures and biomaterials need constant revisions in light of new developments in bone regeneration therapeutics.
